# Balancing the health workforce: breaking down overall technical change into factor technical change for labour—an empirical application to the Dutch hospital industry

**DOI:** 10.1186/s12960-017-0184-5

**Published:** 2017-02-17

**Authors:** Jos L. T. Blank, Bart L. van Hulst

**Affiliations:** 1 Delft University of Technology, Delft, The Netherlands; 2 Erasmus University Rotterdam, Rotterdam, The Netherlands; 3 PO Box 5015, 2600 GA Delft, The Netherlands

## Abstract

**Background:**

Well-trained, well-distributed and productive health workers are crucial for access to high-quality, cost-effective healthcare. Because neither a shortage nor a surplus of health workers is wanted, policymakers use workforce planning models to get information on future labour markets and adjust policies accordingly. A neglected topic of workforce planning models is productivity growth, which has an effect on future demand for labour. However, calculating productivity growth for specific types of input is not as straightforward as it seems. This study shows how to calculate factor technical change (FTC) for specific types of input.

**Methods:**

The paper first theoretically derives FTCs from technical change in a consistent manner. FTC differs from a ratio of output and input, in that it deals with the multi-input, multi-output character of the production process in the health sector. Furthermore, it takes into account substitution effects between different inputs. An application of the calculation of FTCs is given for the Dutch hospital industry for the period 2003–2011. A translog cost function is estimated and used to calculate technical change and FTC for individual inputs, especially specific labour inputs.

**Results:**

The results show that technical change increased by 2.8% per year in Dutch hospitals during 2003–2011. FTC differs amongst the various inputs. The FTC of nursing personnel increased by 3.2% per year, implying that fewer nurses were needed to let demand meet supply on the labour market. Sensitivity analyses show consistent results for the FTC of nurses.

**Conclusions:**

Productivity growth, especially of individual outputs, is a neglected topic in workforce planning models. FTC is a productivity measure that is consistent with technical change and accounts for substitution effects. An application to the Dutch hospital industry shows that the FTC of nursing personnel outpaced technical change during 2003–2011. The optimal input mix changed, resulting in fewer nurses being needed to let demand meet supply on the labour market. Policymakers should consider using more detailed and specific data on the nature of technical change when forecasting the future demand for health workers.

## Background

One of the health policies on which the Organisation for Economic Co-operation and Development (OECD) focuses is an adequate planning of the health workforce. According to the OECD [49], well-trained, well-distributed and productive health workers are crucial for ensuring access to high-quality, cost-effective healthcare in OECD countries. The importance of health workforce planning is acknowledged by European policymakers with the Joint Action on Health Workforce Planning and Forecasting, a 3-year project, financed by the European Commission, aimed at improving the capacity for health workforce planning and forecasting by supporting European collaboration. The challenge for policymakers is to achieve a balance between a shortage and a surplus of health workers. Training too many health professionals leads to unemployment and is expensive, since this implies unnecessary cost for training. On the other hand, training too few health professionals leads to access problems and delayed treatment, possibly with severe consequences. There is no short-run solution to a shortage of health professionals, since the supply of health professionals is rather inelastic. Training health professionals can take a couple of years, depending on the specific profession.

In order to make adequate forecasts of the demand for health professionals, workforce planning models are used. Aside from the demand side aspects of these models, knowledge of the production structure and productivity is of great relevance. In particular, how shifts in healthcare demand are related to the required inputs and how technological changes affect the demand for inputs should be well addressed in these models. To be more specific: productivity changes over time. Productivity growth might bridge a possible gap between demand for and supply of the health labour workforce. Therefore, incorporating productivity growth in planning models seems an obvious move. In practice, however, this is easier said than done.

Productivity is the relationship between one or more inputs and one or more outputs that can be produced with these inputs. Measuring the productivity in the healthcare sector means relating inputs (e.g. physicians, nurses, medical equipment) to outputs (e.g. number of doctor consultations, number of hospital discharges). Basically, the concept of productivity is quite simple; however, Evans et al. [26] point out that ‘the concept of “productivity” is very simple in principle, but rather slippery to pin down in practice’.

The measurement of productivity and productivity growth in healthcare is complicated by the fact that the nature of production is multiple-output, multiple-input. A patient attending a primary care centre may first be examined by a nurse and then, depending on the nature of his disease, be referred to a doctor. Treating a patient in a hospital requires the use of several inputs, such as services professionals (doctors, nurses), material supplies (bandages, pharmaceutics) and capital (beds, medical equipment). To make things more complicated, each patient requires a tailored mix of inputs. We are therefore faced with a multi-input, multi-output production process.

Still, we want to relate inputs to outputs. For that purpose, the production process can be represented through a production or cost function. The amount of required inputs—such as doctors, nurses, material and capital and the corresponding costs—are a function of different outputs, such as the number of hospital discharges and outpatients. Various output combinations (i.e. case mix of patients) influence the amount of inputs required. Productivity might change over time, implying that the relation between inputs and outputs changes. For example, by working smarter or using advanced equipment, the same amount of outputs can be produced with less inputs.

Ono et al. [50] reviewed the workforce planning models of 18 countries and concluded that productivity growth is a neglected topic in workforce planning models. In some of the models, productivity is explicitly included, but mostly as an arbitrary assumption. Other models simply ignore the possibility of productivity growth, and workforce forecasts are directly related to expected changes in service demand. We can illustrate the arbitrary assumptions with two examples: Health Workforce Australia assumes in one of its scenarios a 5% productivity gain for doctors and nurses between 2010 and 2025, without specifying the sources of this productivity gain [32], and the Canadian Nurse Association assumes a 1% productivity growth per year between 2007 and 2022 [19]. This shows that productivity growth is assumed rather than based on empirical estimates.

At the same time, there is huge amount of research available that addresses the topic of productivity and productivity growth. Total factor productivity (TFP) and its decomposition is a popular research topic in the productivity literature (see, e.g. [7, 24, 31, 39]). The central issue in this type of research is breaking down TFP growth into changes in scale, pure technical and allocative efficiencies, and technical change, based on the seminal work of Diewert [24]. Based on his earlier work, research has been extended to cover a myriad of topics and methodological advances (see, e.g. [6, 48]). As will be shown later, these techniques have also been extensively applied to hospitals.

TFP is a general measure that applies to the aggregate of all inputs, whereas our interest is in the productivity of individual inputs, and especially the productivity of the workforce. It is therefore questionable whether it is appropriate to apply a general measure of productivity growth to all inputs. Each input will have its own specific productivity development. Thus, TFP has to be broken down into productivity for specific inputs. A breakdown into the main categories labour, material supplies and capital is obvious, but a more detailed breakdown might be preferred—especially for labour, as for health professionals, the supply of labour is inelastic, while this is far less the case for auxiliary personnel. Despite the increase in literature on TFP, far less attention has been paid to breaking down technical change into productivity measures for individual inputs.

The limited attention paid to the productivity change of individual inputs is striking, since various innovations—such as improved working conditions, absenteeism policy, education and other forms of human resource management—are aimed at increasing labour productivity. From a policy point of view, this is an intriguing situation, since it may provide an opportunity to establish the effects of the aforementioned human resource management measures on productivity. More generally, innovations are most likely to be linked to a particular input, such as an improved use of floor space (capital) or less waste of materials. This paper therefore focuses on the partial factor productivity changes that are directly related to technical change and are controlled for the influences of changes in output and input prices on partial productivity. This results in what we call factor technical change (FTC) for each input. We establish these FTCs from an integral framework in such a way that the derived measures are completely consistent with overall technical change.

This paper is organized as follows. The ‘Methods’ section introduces and explains the concept of FTCs. The more technical derivation of FTCs is included in the Appendix. The next section gives an application of the model to the Dutch hospital industry and discusses the Dutch hospital industry, data and estimation results. The ‘Discussion’ and ‘Conclusions’ sections conclude.

## Methods

### Productivity analysis of hospitals

There is a large body of literature on the efficiency and productivity of hospitals. Hollingsworth [36] identifies 165 journal articles and book chapters on the efficiency and productivity of hospitals. Hollingsworth and Street [37] note that the popularity of efficiency and productivity studies was increasing, most likely as a result of increased demand for adequate information for decision makers and of lower research barriers resulting from the improved availability of data and easy-to-use software. The numerous efficiency and productivity studies have in turn led to several systematic review studies (see for example [35–37, 46, 47, 55, 56, 62]). All these efficiency and productivity studies concentrate on applying data envelopment analysis [20] or stochastic frontier analysis [1, 44].

Most studies on hospitals focus on the effects of environmental pressures on efficiency and productivity, such as payment systems, competition and property rights [3, 9, 10, 29, 30, 45, 52, 54, 57, 61]. Other studies focus on economic phenomena, such as economies of scale, economies of scope, economic behaviour and expense preference [12, 15, 16, 23, 41]. The influence of managerial and organizational aspects, such as outsourcing or the size of departments, is the central focus in a number of other studies [2, 13, 34, 42, 43].

Only a limited number of studies focus on estimating hospitals’ technical change [8, 17, 18, 30, 38]. Of these studies, only Blank and Vogelaar [18] and Blank and van Hulst [17] explicitly refer to the different natures of technical change (i.e. input- and/or output-biased). Even less attention is paid to the increase in labour productivity. From a policy point of view, however, labour productivity growth is at the centre of attention. With an aging population, the demand for healthcare is growing, while a decreasing labour force makes the labour market tight. An interesting exception is an article by Ozcan et al. [51] that addresses the development of labour efficiency in hospitals with an explicit reference to the labour market for hospital personnel.

### Factor technical change

This paper focuses on the partial factor productivity changes that are directly related to technical change and are controlled for the influences of changes in output and input prices on partial productivity. This results in what we call factor technical change (FTC) for an input.

It is important to note that FTC differs from partial factor productivity growth. Factor productivity growth is the change of output divided by the volume change of an input. The volume change of an input depends not only on the change in output but also on technological change and substitution effects. These substitution effects are a result of changes in relative prices, which change the optimal input mix. If we take, for example, increasing wages with the price of capital fixed, it makes sense to substitute capital for labour. This enhances labour productivity, as less labour is used. Similar effects might result from changing output levels and mix. The FTC measures the extent to which factor productivity changes due to technological change and can be regarded as factor productivity after controlling for output levels and input prices.

The approach presented here is strongly connected with the research on the various types of technical progress or digress. This type of research goes back to Schumpeter [58] and also Hicks [33] and Solow [60]. Generally, three types of technical change are distinguished in the literature:

- Technological change is called Hicks neutral if the ratios of the various marginal outputs to inputs are unchanged due to technological change. Note that, in that case, technological change and FTC will be equal for all inputs.
- Technological change is called input-biased if the relationship between input demand and input prices is affected by the introduction of new technologies. Putting it differently, this implies that input demand, controlled for changes in outputs and input prices, changes in time due to technical change. Note that FTCs may differ accordingly.
- Technological change is called output-biased if the relationship between input demand and levels and mix of outputs is affected by the introduction of new technologies.

A combination of input-biased and output-biased is also possible.

**Fig. 1:**
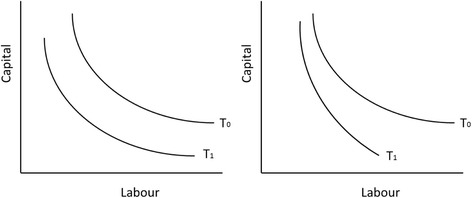
An example of neutral (*left*) and input-biased (*right*) technological change

Figure 1 shows an example of neutral technological change and input-biased technological change. The figure shows isoquants that represent combinations of labour and capital and can produce the same amount of output. Isoquant *T*_0_ represents the original technology, and *T*_1_ is the technology that results after technological change. In the left-hand panel, isoquants *T*_0_ and *T*_1_ are parallel and the technological change is said to be neutral. At technology *T*_1_, less inputs are required to produce the same amount of outputs, at the same time, the ratios of marginal output to inputs are unchanged. In the right-hand panel, the technological change is input-biased; there is no parallel shift. Therefore, not only less inputs are required but also the ratios of marginal output to inputs change. In this particular example, it is advantageous substituting capital for labour.

The example shows that if technological change is input-biased, substitution will take place. However, as mentioned in the introduction, input-biased technological change is not the only reason to substitute: changes in relative prices may also lead to substitution. FTC is a measure that is corrected for these price effects; hence, FTC is a pure effect of technological change.

The Appendix of this paper shows how FTCs are derived from technical change in a cost model framework. In a cost model, separate input demand equations can be derived, which provide the necessary information for FTC. The method is elaborated for a so-called translog cost function (see [21]). This is a flexible mathematical form that allows for many different shapes of the cost function. It can therefore also handle the different types of technical change. It is shown that in the case of a translog cost function, the FTC of input *n* equals total technical change and a correction parameter for the specific input.

## Application to the Dutch hospital industry

### The Dutch hospital industry

This section presents the results of the model applied to the Dutch hospital industry. We start with a brief description of the Dutch hospital industry, as some choices made in the application are a result of the specific Dutch context and data availability. Dutch hospitals can be divided into three types, namely general hospitals, teaching hospitals and specialist hospitals. This paper concerns only the general hospitals because the characteristics of teaching and specialist hospitals differ too much from those of general hospitals. Including teaching and specialist hospitals would imply adding heterogeneity to the data. Moreover, the vast majority of hospitals in the Netherlands are general hospitals, accounting for about 80% of all hospital beds and almost 70% of all hospital costs. A general hospital is a concentration of facilities for diagnosing, treating and nursing patients; other activities include the training of physicians and nurses.

In modelling the costs of hospitals, we have to pay special attention to the costs of physicians. Some physicians are employed by hospitals, but most are entrepreneurs who cooperate with hospitals. The costs and funding of physicians and hospitals are strictly separated. One drawback of this arrangement is that the data on physicians are incomplete and including physicians in an empirical application requires special attention. This also means that data on costs should be corrected for the costs of the physicians who are employed by hospitals.

### Data

#### General

All hospitals are obliged to present annual reports containing information on costs, output and some specific hospital characteristics. Besides the annual reports, there is a yearly survey containing information on specific inputs and some other hospital characteristics. Data from the annual reports are freely available; the additional data from the survey was obtained from the Dutch general hospital association (NVZ). The data on the period 2003–2011 from both sources were combined into one dataset suitable for analysis. The dataset was checked for observations with unreliable or missing data, which resulted in the removal of 15 observations. Furthermore, there were a couple of mergers during the period of analysis. As a result of mergers and the removal of observations, the number of observations for each year are unequal. The final dataset comprises 672 observations, about 75 hospitals per year.

#### Outputs

The output of hospitals is generally measured by the number of admissions and outpatients. However, only including admissions and outpatients visits as output does not account for the heterogeneity of the production of hospitals. Depending on the data availability, models include different case mix and quality indicators. Rosko and Mutter [56] provide an overview of the following applied case mix and quality indicators: proportion of inpatient days in intensive care units, births as a proportion of admissions, intra-DRG severity of illness index, number of high-technology services, teaching status, full-time equivalent resident physicians, joint Commission on Accreditation of Healthcare Organizations accreditation, number of board-certified medical staff per bed, existence of a transplant programme, risk-adjusted mortality rates, risk-adjusted patient safety event rates and patient burden of illness. A frequently used indicator of case mix is a hospital’s teaching versus non-teaching status [36, 56].

Here, we apply a hedonic index [40] that accounts for the characteristics of the hospital. The hedonic index contains the following elements: relative size of the surgery and orthopaedic departments (measured by the number of physicians), expected length of stay (based on the mix of specialties available in the hospital), number of intensive care (IC) beds, presence of a psychiatric ward, presence of neurosurgery and presence of cardiothoracic surgery. The hedonic index is a straightforward tool that accounts for case mix differences amongst hospitals. The admissions included in the cost function are weighted by the hedonic index, which credits hospitals with a more severe case mix in accounting for cost differentials.

Aside from the number of patients and the case mix, hospitals may also differ in health outcomes. Accounting for health outcomes is hard, since good, consistent, quality data are mostly unavailable. Quality data are now collected in the Dutch hospital industry. For example, Baines et al. [5] look at the adverse event rate between 2004 and 2008 in Dutch hospitals. However, the authors summarize the limitations and emphasize that a comparison between the 2 years of analysis must be made with caution. Furthermore, the study is based on a sample of hospitals for only 2 years, which is insufficient for our case. So although data are available, they are insufficient for our analysis. Besides, we argue that in the Dutch context, the quality differences are not an issue since the inspectorate of healthcare supervises the sector intensively. Physicians and hospital managements follow strict protocols, guaranteeing a high minimum standard.

In addition to treating patients, hospitals perform research activities and other services. This output is measured by the revenues that these activities and services generate; the revenues are deflated (i.e. adjusted for price effects over time).

A shortcoming of the measurement of output is that health outcomes and the number of patients treated are not synonymous. In other words, output indicators such as admissions and outpatients are only a proxy for health outcome. Porter [53] makes a strong argument for the use of health outcomes. Although health outcomes are preferable, data on health outcomes are not available. At the same time, as mentioned earlier, there are reasons to assume that the quality of Dutch hospital care does not vary very much, as quality is constantly monitored, for instance by the health inspectorate, patient associations and the media, and is subject to interventions that improve quality. Moreover, Blank and van Heezik [14] argue that for the Dutch hospital industry, quality has improved at the macro-level. Since an eventual improvement in quality is neglected, this implies that estimated FTCs might have a downward bias. Furthermore, it should be noted that the use of health outcomes is not a restriction of the model that calculates the FTCs: it is shortcoming of workforce planning models in general. The calculation of FTCs would be similar where data on health outcomes are available.

#### Inputs and input prices

A common classification of inputs is labour, materials and capital. Since our aim is to obtain the FTC for inputs, it makes sense to use a more detailed classification of the main input categories. Moreover, we want to distinguish inputs that are rather homogenous and have some characteristics that make them different from the other inputs. At the same time, we want to be sparing with the number of categories, since each additional category requires the estimation of several extra parameters. Besides the classification of input categories, it is also limited by the data that are available.

Classification of the labour categories is done on the basis of cost homogeneity and matching of professions. Dutch hospitals use a standardized job classification system that includes both main categories and subcategories of personnel. These categories and subcategories are included in our data and allow the matching of professions. The result is the following four labour categories:

- Management and clerical staff
- Nursing personnel
- Paramedic personnel
- Auxiliary personnel (e.g. maintenance, kitchen staff, and security and cleaning personnel)

For each of the four labour categories, there are data on the costs and the number of full-time equivalents. Unit values are used as prices. For each category, prices are calculated by dividing the corresponding costs and full-time equivalents. The implicit assumption made here is that prices are exogenous for hospitals, and all price variation comes from exogenous factors. In the sensitivity analysis, we will pay attention to this assumption and present an alternative.

One labour input is missing: physicians. As mentioned, many physicians are entrepreneurs, and consequently, data on the costs of physicians are not included in the hospital data. Therefore, physicians are omitted from the model. In the sensitivity analysis, a circumventing construction is used to include physicians in the model.

For the material costs, we distinguish two categories: costs that are directly related to patients (e.g. medical supplies, food and hotel costs) and other material costs (e.g. energy and general costs). We acknowledge that a more detailed categorization for medical supplies, especially one including a separate category for medicine, would be more sophisticated. However, a more detailed categorization is limited by the lack of data; besides, we want to be sparing. Since there is no natural unit of measurement for material supplies, a circumventing construction is used. For both categories, we use the price indices calculated and published by Statistics Netherlands. Price indices vary only over time; for our purpose, this is adequate since there is no reason to assume variations in prices of materials between regions. For both main categories of materials, we have subcategories for which we have data on the costs at the hospital level and a price index at the national level. The price indices for both main categories are constructed for each hospital as the weighted average of the price indices of the subcategories. Price indices of subcategories are weighted by matching hospital-specific cost shares.

For capital, we use only one input category, as it is hard to find appropriate measures for capital. Capital refers to the capital assets such as buildings and medical equipment. In the available data, there are some indicators of the volume of capital stock. The volume of capital is measured as a weighted aggregate of beds, IC beds, psychiatric beds, square metres and the number of linear accelerators and cobalt units. The weights for each capital stock indicator are obtained by a regression of the capital cost on the indicators. The price of capital is defined as a unit value, derived from capital costs and the aforementioned volume of capital.

Table 1 contains the descriptive statistics for the variables used in the cost function. The descriptive statistics are related to 2011, the latest year in the dataset.

**Table 1:** Descriptive statistics, Dutch general hospitals 2011 (*N* = 69)

Variable	Mean	Standard dev.
Admissions	45 055	20 834
Outpatients	76 967	29 281
Other revenues (× million €)	14.7	10.8
Surgery and orthopaedics (%)	11.7	2.4
Psychiatric beds per 1000 admissions	0.27	0.40
IC beds per 1000 admissions	0.23	0.09
Expected length of stay	3.3	0.3
Neurosurgery (%)	0.8	1.9
Cardiothoracic surgery (%)	0.3	0.9
Price management and clerical staff	53 898	2 799
Price nursing personnel	54 476	1 297
Price paramedic personnel	90 142	6 662
Price auxiliary personnel	40 491	1 637
Price patient-related material costs	1.12	0.005
Price general material costs	1.16	0.008
Price capital	2.1	0.64
Total cost (× million €)	148.3	77.9
Cost share management and clerical staff	0.10	0.02
Cost share nursing personnel	0.34	0.04
Cost share paramedic personnel	0.03	0.02
Cost share auxiliary personnel	0.09	0.02
Cost share patient-related material costs	0.21	0.03
Cost share general material costs	0.13	0.03
Cost share capital	0.09	0.02

### Model estimation

The model is specified as a translog long-run cost function and corresponding cost share equations, which are derived from the cost function (see Equations 7 and 8 in the Appendix). The specification of the model includes three output variables (with a correction for case mix), seven input price variables and year dummies to measure technical change. The reason these variables were chosen was discussed in the previous section.

Since we are dealing with a relatively large number of cross-sectional units and a limited number of periods, we ignore the fact that we are dealing with panel data (with respect to intra-firm correlations). Not much harm is done here, since the between variance is far more relevant for the estimation than the within variance. We therefore pool all the data in one dataset and control for the time varying effects by including a technology variable. For reasons of convenience, technical change is often modelled by using a time trend, implicitly assuming that technical change is a rather smooth process in time.

Previous research on Dutch hospitals [18] shows that technical change appears to be shock-wise, that is technological change is not a smooth process but varies from year to year. The researchers argue that including a time trend in a cost function model is a rather restrictive way of incorporating technical change (ibid.). We follow the time trend approach, since that approach is in line with the way that the model is mathematically derived. The shock-wise approach, with year dummies instead of a time trend, is included in the sensitivity analysis.

Homogeneity of degree one in prices and symmetry are imposed by adding restrictions to the model. Aside from these imposed theoretical requirements, a few other requirements also need to be fulfilled, such as monotonicity and concavity in input prices [27]. These requirements can be tested posteriorly. An estimated cost function is monotonic in input prices if the fitted cost shares are positive. Concavity can be tested by exploring necessary and sufficient conditions for concavity.

The parameter estimates of the full model are included in the Appendix. Almost three quarters (74%) of the parameter estimates are significant at the 5% level. Estimated parameters also have the expected signs. Since the fitted cost shares are positive for all firms, the theoretical condition for monotonicity is satisfied for all inputs. A necessary condition for concavity of the cost function is that the own partial elasticities of substitution be less than zero for all inputs. This necessary condition for concavity of the cost function holds for all the inputs. A sufficient condition is that the matrix of partial elasticities of substitution is negative semi-definite. A matrix is negative semi-definite if all eigenvalues are less than or equal to zero. The eigenvalues of the matrix of partial elasticities of substitution can be calculated for each observation. The matrix of partial elasticities of substitution is negative semi-definite for 87% of the observations; 13% of the observations fail the sufficient conditions. For these observations, the sufficient condition is too tight.

A quick inspection of the estimated parameters of the output variables shows that the average hospital faces diseconomies of scale (∑*b*_*m*_ = 1.11 > 1). For the average hospital, a 1% increase in outputs corresponds to a 1.11% increase in total costs. Furthermore, the cost flexibilities, namely the responsiveness of the costs to changes in output, can be calculated for the individual hospitals.

Also note that the cross parameters of time and input prices (*j*_in_) are significant in six out of seven cases. These parameters are a key element in the formula of the FTCs (see Equation 11). Furthermore, we tested the model against the alternative model with the cross parameters of time and input prices set to zero. Based on the likelihood ratio test, the alternative model is overwhelmingly rejected (likelihood of 11 712 and 11 672). We therefore find that technological change is input biased.

## Results

Applying (10) and (11) and using the parameter estimates yield the following overall technical change and FTCs per type of input (see Table 2).

**Table 2:** Index factor technical change per type of input (2003 = 100)

	Overall	Management and clerical staff	Nursing personnel	Paramedic personnel	Auxiliary personnel	Material, patients	Material, general	Capital
2003	100.0	100.0	100.0	100.0	100.0	100.0	100.0	100.0
2004	102.8	103.3	103.2	105.4	104.8	101.5	100.8	103.5
2005	105.6	106.6	106.4	110.2	109.9	102.9	101.4	107.1
2006	108.4	109.9	109.6	115.2	115.3	104.3	102.0	110.6
2007	111.2	113.3	112.9	120.6	120.9	105.6	102.7	114.0
2008	114.0	116.8	116.2	124.4	126.9	107.0	103.3	117.7
2009	116.8	120.2	119.5	128.9	133.1	108.4	103.8	121.1
2010	119.4	123.4	122.7	133.3	139.9	109.6	103.9	124.9
2011	122.1	126.7	125.9	138.0	146.8	110.7	104.2	128.2

Table 2 shows that between 2003 and 2011, the technical change was 22.1%, which is an annual technical change of 2.8%. The FTC per type of input differs from this measure. The FTCs for nursing personnel and management and clerical staff are slightly higher than the general productivity measure: they show an increase of 26 and 27%, respectively, between 2003 and 2011. The increase of the FTC for capital is even a little higher than this, at 28%. Significant differences between FTC and total technical change exist for both paramedic personnel and auxiliary personnel. The FTC for auxiliary personnel is substantially higher than the FTC of all other inputs: between 2003 and 2011, the FTC increase is 47%. In the past period, hospitals have been reducing the costs of auxiliary personnel: during the period of analysis, the volume of auxiliary personnel dropped by roughly 15%. For paramedic personnel, there is also an increase of FTC that is higher than total technical change, namely 38%. We should keep in mind that paramedic personnel comprise a rather small group on which fluctuations can have a large impact. Most notable is the development of the FTCs of medical supplies and material supplies: both lag behind. The lack of FTC gains should not be regarded as a bad thing; however, it merely reflects the increased or decreased relative importance of an input due to technical change.

We performed a couple of sensitivity analyses to gain an insight into the reliability of the results. In the main analysis, a detailed classification of the inputs is introduced. In this sensitivity analysis, we use the common classification into three inputs: labour, materials and capital. The results are shown in Table 3.

**Table 3:** Index factor technical change per type of input (2003 = 100)

	Overall	Labour	Materials	Capital
2003	100.0	100.0	100.0	100.0
2004	102.6	103.3	101.2	103.3
2005	105.2	106.7	102.2	106.6
2006	107.8	110.1	103.3	109.8
2007	110.3	113.5	104.3	112.9
2008	112.8	116.9	105.2	116.3
2009	115.2	120.3	106.1	119.3
2010	117.6	123.8	106.9	122.6
2011	119.8	127.1	107.6	125.5

Table 3 shows that aggregating inputs has a minor impact on the results. The estimated total technical change is a little lower when the inputs are aggregated. The annual difference is about 0.3 of a percentage point and accumulates to a difference of 2.2 percentage points for the whole period. Capital is the only input that is not further aggregated in this sensitivity analysis. For this input, the difference of FTC is in line with the differences for total technical change. It is only a little lower than the model with seven inputs.

For labour, the sensitivity analysis demonstrates the added value of disaggregating: the FTC of labour as an aggregate is a crude measure for the FTC underlying labour categories. For most labour categories, deviations are small, but for auxiliary personnel, the deviation is huge. For this category, the development has been quite different from that of the other labour categories. For materials, the FTC lies between the FTC of both underlying categories.

As mentioned, the data do not allow us to directly distinguish physicians as an input. In this sensitivity analysis, we use a circumventing approach. Data on the costs of independent physicians are obtained by multiplying the average profits of independent physicians by the number of physicians (since independent physicians have no wages, profits are good indication). The national bureau of statistics has data on the average profits generated by 15 specialisms. For physicians on the payroll of hospitals, we take a fixed amount based on the collective labour agreement for these physicians. By doing so, we have rather crude estimates of the costs and prices of physicians that can be incorporated in the model. The data are sufficient for this sensitivity analysis; however, analysis of the FTC of physicians requires more accurate and detailed information. Table 4 shows the results of the model when physicians are included.

**Table 4:** Index factor technical change per type of input (2003 = 100)

	Overall	Management and clerical staff	Nursing personnel	Paramedic personnel	Auxiliary personnel	Material, patients	Material, general	Capital	Physicians
2003	100.0	100.0	100.0	100.0	100.0	100.0	100.0	100.0	100.0
2004	102.6	103.2	103.1	103.6	104.8	101.3	100.8	103.6	101.7
2005	105.1	106.4	106.2	107.1	109.8	102.5	101.6	107.1	103.3
2006	107.5	109.5	109.3	110.5	115.1	103.6	102.2	110.5	104.8
2007	109.9	112.7	112.4	114.2	120.6	104.7	102.8	113.8	106.3
2008	112.2	115.9	115.5	117.5	126.5	105.7	103.4	117.4	107.8
2009	114.4	119.1	118.6	120.7	132.7	106.6	103.7	120.6	109.3
2010	116.7	122.0	121.6	122.9	139.2	107.6	104.0	124.4	110.2
2011	118.9	125.1	124.7	125.6	146.0	108.6	104.4	127.6	110.9

Including physicians in the model results in less total technical change. This is because the FTC of physicians lags behind (total technical change is a weighted average). Furthermore, we notice a remarkable difference for the FTC of paramedic personnel, which drops by 12.5 percentage points. This suggests that the FTC calculated for paramedics is less robust than other FTCs and should be interpreted with care. Note that for all other inputs, the effect of including physicians are marginal; for example, FTC for nursing personnel is 3.2% in the model without physicians versus 3.1% in the model including physicians (see also Table 5).

**Table 5:** Average annual total technical change and FTC for nursing personnel

	Total	Nursing personnel
Base model	2.8	3.2
Aggregated inputs^a^	2.5	3.4
Physicians included	2.4	3.1
Regional prices	2.1	2.4
Shock-wise technological change	2.8	3.4

^a^The FTC presented for nursing personnel is the FTC for labour

Another sensitivity analysis is performed by defining the prices of labour differently. As stated, we use unit values, assuming that all price variations are exogenous. Although it is common practice to use unit values, there is something to say for an approach that assumes partly endogenous price variation. The Dutch labour market is regulated; therefore, in the case of wages, equal exogenous conditions are plausible. However, some regional variation might be expected as there are differences between regional labour markets. We now drop the assumption of complete exogenous price differences and assume that price differences are a result of regional exogenous factors and endogenous factors. We therefore calculate annual regional prices, or market price, for labour as the average price in a region in a year. Differences between the regional price and the actual observed price of a hospital are ascribed to endogenous factors. The model is estimated with the regional prices.

Not only do the results for the FTCs change but also the statistics of the model change. Most noticeable is that the sufficient condition for concavity fails for all observations (either the model is invalid or possibly the sufficient condition for concavity is too tight). For the FTCs, we observe clear changes. For instance, the FTC for management and clerical personnel is 10 percentage points higher, while the FTC of nurses drops by 7 percentage points. Other FTCs also change to some extent. One possible explanation for the different results is that the FTCs, as calculated in the base model, include trade-offs between the price of inputs and their productivity. For example, a hospital can decide to hire not only more expensive but also more experienced staff, assuming that higher wages pay off in higher productivity by more experienced staff. The sensitivity analysis corrects for these trade-offs since it excludes endogenous price effects. From a model perspective, the changes are due to changes in the parameters estimated for the interaction term of time and inputs (*j*_11_ *–j*_17_). However, we must refrain from drawing any bold conclusions, since we have some doubts about the statistical properties of the model (concavity). What we can conclude is that the outcomes of the model are sensitive to the definition of input prices.

Finally, we test a model in which the time trend is replaced by a so-called technology index, represented by a set of dummy variables for each year and weighted by (estimated) weights [18]. Based on the log likelihoods (11 711 for the trend model and 11 716 for the shock-wise model), we cannot conclude that the shock-wise model has to be preferred over the trend model. The big difference between the trend model and the shock-wise model is that in 2004, the latter model shows a higher productivity growth, while in 2010, the productivity is less. Aside from that, there are a couple of small deviations. As expected, over the whole period of analysis, the deviations balance each other out. For the FTCs, there are yearly differences that are in line with the differences observed for total technical change. For the whole period, the deviations are rather small, namely 1 percentage point for most inputs, with a maximum of 1.6 percentage points.

In general, the sensitivity analysis supports the results of the base model. Table 5 summarizes the results of the sensitivity analysis and shows the average annual technical change and the average annual FTC for nursing personnel. Our focus in the table is on nursing personnel since this is the category that is relevant from a policy perspective. The absolute magnitude of the differences in average annual technical change is small. An exception is the model with adapted prices, where the differences are noticeable. The sensitivity analysis shows that some care has to be taken when making statements about the magnitude of FTC.

## Discussion

The computation of FTCs in a cost function framework does justice to the multi-input, multi-output production process of hospitals, without using ex ante weights (‘before the event’ weights) for the products. Furthermore, FTCs are consistent with the concept of technological change. They incorporate input-biased technological change and therefore take into account that changes in productivity might vary between inputs. FTCs purely measure the effect of technological change; it is adjusted for substitution effects resulting from price effects.

A possible shortcoming of empirical modelling of FTCs is the availability of suitable data. In general, the measurement of healthcare output will only be a proxy of better health outcomes; that is due to data availability, most of the time output will be defined in terms of quantity and volume (and not quality and value). Furthermore, modelling requires parsimony in the number of parameters, requiring some level of aggregation. However, we should bear in mind that traditional workforce planning use similar data and will therefore face the same shortcomings. At the same time, empirical modelling of FTCs can incorporate data, if available, that does more justice to health outcomes or quality.

An empirical model is applied to the Dutch hospital industry during the period 2003–2011. The empirical model uses a translog cost function with three outputs and an additional case mix indictor and seven inputs. The results of the empirical model show that due to technical change, productivity increased by an average of 2.8% a year in the Dutch hospital industry during the period 2003–2011. The results also show that technical change is input-biased and that FTC differs amongst the various inputs. The FTC of nursing personnel increased at a higher rate (i.e. 3.2% per year) than total technical change. One of the interesting aspects of the tremendous change in productivity is that there appears to have been a period during which Baumol’s cost disease [11] did not afflict Dutch hospitals. The Baumol effect connotes that in labour-intensive sectors, such as nursing, there is little or no productivity growth. The results here indicate that the opposite is true.

Furthermore, the results are not limited to Dutch hospitals; there are several studies that find substantial growth of TFP for hospitals. For example, Azevedo and Mateus [4] find 7% growth of TFP during 2003–2009 for Portuguese hospital, Daidone and D'Amico [22] find 14% growth of TFP for Italian hospitals during 2000–2005, and Farsi and Filippini [28] find 14.3% growth of TFP for Swiss hospitals during 1998–2001. And although growth of FTP does not correspond one to one with FTC of nursing personnel, it is reasonable to assume that decomposition of TFP in these studies would show also substantial growth of FTC. Indicating that there are more studies that find that there is sometimes a cure for Baumol’s cost disease.

The results for the FTC of materials are interesting. For materials, we distinguished two categories: one category of costs that are directly related to patients and one more general category. For both categories, we observe that the FTC lags behind with an annual average increase of 1.3 and 0.5%, respectively. But we have to be careful with interpretation here: since prices used for materials are a crude approximation, we can speculate that this is a compound result of two opposite forces. In the period of analysis, we observe a remarkable decline in the average length of stay.^1^ Since one of our output indictors is the number of admissions, it is clear that a shorter length of stay is an important explanation of increased productivity (fewer nursing days per admission). At the same time, an admission becomes more intensive (more materials are used per nursing day). For materials, this apparently results in only small increases in FTC, suggesting that relatively more of these inputs have been used.

## Conclusions

Policymakers use workforce planning models to get information on future labour markets and adjust policies accordingly, as both a shortage and a surplus of health workers are unwanted. One pitfall of workforce planning models is that productivity growth is often neglected. Sometimes, it is completely ignored, and sometimes, an arbitrary growth rate is assumed (see, e.g. Ono et al. [50]). Productivity changes need to be incorporated in the planning, since future demand for health professionals will probably be tempered by increased productivity. In this paper, we presented a framework that breaks down technical change into FTC, a productivity measure that is consistent with technical change and accounts for substitution effects. FTCs can be used to include productivity growth in health workforce planning models and improve forecasts of the demand for health professionals.

The FTC of input *n* is defined as the relative change in usage of input *n* at a given level of output and given input prices due to technical change. FTC differs from partial factor productivity growth: it is a pure measure of the effect of technological change rather than a ratio of output and input that also includes substitution effects. The FTC of an input equals the total technical change and a correction parameter for the specific input. Growth in FTC will reduce shortages on the labour market. An empirical application to the Dutch hospital industry show substantial growth of FTC for nursing personnel. And this is good news as the European Commission estimates, based on several assumptions and hypotheses, a potential shortfall of around 1 million healthcare workers in the EU by 2020 [25].

Policymakers can apply FTCs in human workforce forecasting models by using forecasts of FTCs. This, of course, raises the matter of how to get a forecast of FTCs. A practical and straightforward solution is to use the FTC realized in the past. This solution is flawed, however, as past results are no guarantee of future results. At the same time, it is an improvement compared to models that ignore productivity or assume some level of productivity growth without any empirical evidence. Furthermore, from the empirical application to the Dutch hospital industry, we see that during 2003–2011, there were only small fluctuations. This implies that a forecast based on the past for this period would have worked well.

## Appendix

### A model for factor technical change

#### Theoretical model

We define the FTC of input *n* as the relative change in usage of input *n* at a given level of output and given input prices due to technical change. For reasons of convenience, the decomposition is based on a cost function framework. Furthermore, we assume that the firm is a cost-minimizing decision-making unit.

Assume the cost structure is represented by a well-defined cost function:

1 $$ C= c\left( y, w, T\right) $$

With:

*C* = total cost;

*y* = output (vector of dimension *M*);

*w* = input prices (vector of dimension *N*);

*T* = time (reflecting technological change).

And *c*(*y*, *w*, *T*) is a twice differentiable function with respect to *w* and *T*, which satisfies the requirements concerning monotonicity and concavity (see, e.g. [27]).

By definition, the volume of input *n* is given by:

2 $$ {x}_n\equiv \frac{S_n\cdot C}{w_n}\kern1em  n=1,.., N $$

with:

*X*_*n*_ = volume of input *n*;

*S*_*n*_ = cost share *n*;

*W*_*n*_ = input price for input *n*.

For reasons of convenience, we rewrite Equation (2) in logarithms:

3 $$ \ln {x}_n= \ln {S}_n+ \ln C- \ln {w}_n $$

The total differential of (3) with respect to *T* yields:

4 $$ \frac{d \ln {x}_n}{ d T}=\frac{\partial \ln {S}_n}{\partial T}+\frac{\partial \ln C}{\partial T}-\frac{\partial \ln {w}_n}{\partial T}\kern2.75em  n=1,.., N $$

Input prices are exogenous. Furthermore, we assume that input prices do not depend on technological change or any other time-related variable; consequently, the last term on the right-hand side drops from the equation.

Since we further assume that the firm is cost minimizing, Shephard’s lemma holds [59]. From this, we derive Equation (5) for the identification of the optimal input demands:

5 $$ {S}_n=\frac{\partial \ln C}{\partial \ln {w}_n}\kern2.5em  n=1,.., N $$

Substituting (5) into (4) yields:

6 $$ \frac{d \ln {x}_n}{ d T}=\frac{1}{S_n}\frac{\partial \left[\frac{\partial \ln C}{\partial \ln {w}_n}\right]}{\partial T}+\frac{\partial \ln C}{\partial T}\kern2.25em  n=1,.., N $$

which is the basic expression for a FTC. The percentage change in input *n* equals the percentage change in cost due to overall technical change (second term on the right-hand side), corrected for the relative annual change in the corresponding cost share (first term on the right-hand side).

#### Empirical application

We now apply the well-known translog cost function. The cost function model consists of a translog cost function and the corresponding cost share equations.

7 $$ \begin{array}{ll} \ln C={a}_0\hfill & +{\displaystyle \sum_{m=1}^M}{b}_m \ln {y}_m+{\displaystyle \sum_{n=1}^N}{c}_n \ln {w}_n+{\displaystyle \sum_{o=1}^O}{d}_o \ln {z}_o+\frac{1}{2}{\displaystyle \sum_{m=1}^M}{\displaystyle \sum_{m^{\hbox{'}}=1}^M}{b}_{m{ m}^{\hbox{'}}} \ln {y}_m \ln {y}_{m^{\hbox{'}}}\hfill \\ {}\hfill & +\frac{1}{2}{\displaystyle \sum_{n=1}^N}{\displaystyle \sum_{n^{\hbox{'}}=1}^N}{c}_{n{ n}^{\hbox{'}}} \ln {w}_n \ln {w}_{n^{\hbox{'}}}+\frac{1}{2}{\displaystyle \sum_{o=1}^O}{\displaystyle \sum_{o^{\hbox{'}}=1}^O}{d}_{o{ o}^{\hbox{'}}} \ln {z}_o \ln {z}_{o^{\hbox{'}}}\hfill \\ {}\hfill & +{\displaystyle \sum_{m=1}^M}{\displaystyle \sum_{n=1}^N}{e}_{m n} \ln {y}_m \ln n+{\displaystyle \sum_{o=1}^O}{\displaystyle \sum_{n=1}^N}{f}_{o n} \ln {z}_o \ln {w}_n\hfill \\ {}\hfill & +{\displaystyle \sum_{o=1}^O}{\displaystyle \sum_{m=1}^M}{g}_{o m} \ln {z}_o \ln {y}_m+{h}_0 T+{h}_1{T}^2+{\displaystyle \sum_{m=1}^M}{i}_{1 m} T \ln {y}_m\hfill \\ {}\hfill & +{\displaystyle \sum_{n=1}^N}{j}_{1 n} T \ln {w}_n\hfill \end{array} $$

with:

*C* = total costs;

*y*_*i*_ = output *i* (*i* = 1,.., M);

*w*_*i*_ = price of input *i* (*i* = 1,.., N);

*z*_*i*_ = fixed input *i* (*i* = 1,.., O);

*T* = time.

${a}_0,{b}_m,{c}_n,{d}_o,{b}_{m{ m}^{\prime }},{c}_{n{ n}^{\prime }},{d}_{o{ o}^{\prime }},{e}_{m n},{f}_{o n},{g}_{o m},{h}_0,{h}_1,{i}_{1 m},{j}_{1 n}$ parameters to be estimated.

And applying Shephard’s lemma, we find the optimal input demands:

8 $$ {S}_n^0={c}_n+{\displaystyle \sum_{i=1}^N}{c}_{i n} \ln {w}_i+{\displaystyle \sum_{i=1}^M}{e}_{i n} \ln {y}_i+{\displaystyle \sum_{i=1}^O}{f}_{i n} \ln {Z}_i + {j}_{1 n}\  T\kern1.5em  n=1,.., N $$

with:

*S*_*n*_^0^ *= optimal cost share for input n (n = 1,.., N).*

Homogeneity of degree one in prices and symmetry is imposed by putting constraints on some of the parameters to be estimated. In formula:

9 $$ \begin{array}{lll}{b}_{m{ m}^{\hbox{'}}}={b}_{m^{\hbox{'}} m};\hfill &\ {c}_{n{ n}^{\hbox{'}}}={c}_{n^{\hbox{'}} n};\hfill & {d}_{o{ o}^{\hbox{'}}}={d}_{o^{\hbox{'}} o}\kern0.5em ;\hfill \\ {}{\displaystyle \sum_{n=1}^N}{c}_n=1;\hfill & {\displaystyle \sum_{n=1}^N}{c}_{n{ n}^{\hbox{'}}}=0\kern0.5em \left(\forall {n}^{\hbox{'}}\right);\hfill & {\displaystyle \sum_{n=1}^N}{e}_{m n}=0\ \left(\forall m\right);\hfill \\ {}{\displaystyle \sum_{n=1}^N}{j}_{1 n}=0;\hfill & {\displaystyle \sum_{n=1}^N}{f}_{o n}=0\ \left(\forall o\right).\hfill & \hfill \end{array} $$

Differentiating the translog cost function with respect to input price and time results in an expression for the first right-hand side term of expression (6). This is fairly simple, since the translog cost function (7) contains only one term that depends on both input price and time:

10 $$ \frac{\partial \left[\frac{\partial C}{\partial \ln {w}_n}\right]}{\partial T}={j}_{1 n} $$

Substituting (10) into (6) yields:

11 $$ \frac{d \ln {x}_n}{ d T} = \frac{j_{1 n}}{S_n}+\frac{\partial \ln C}{\partial T}\kern1.25em  n=1,\dots, N $$

Equation (11) shows that in the case of a translog cost function, the FTC of input *n* equals the autonomous growth of cost and a correction factor for the extent of input- and output-biased technological change. It can easily be verified that a weighted aggregate of FTCs using cost shares as weights, equals overall technical change $\frac{\partial \ln C}{\partial T}$. This follows from the notion that the sum of *j*_*1n*_ equals zero and the sum of the cost shares (*S*_*n*_) equals one.

Both right-hand side terms of (11) can be elaborated. For the first term, it is possible to substitute *S*_*n*_ with (8). The second term is the first derivative with respect to *T*. In the case of the translog cost function, this yields the following expression:

12 $$ \frac{\partial \ln C}{\partial T} = {h}_0+{h}_1{T}^2+{\displaystyle \sum_{m=1}^M}{i}_{1 m} \ln {y}_m+{\displaystyle \sum_{n=1}^N}{j}_{1 n} \ln {w}_n $$

#### Model estimation

The model is estimated as a multivariate regression system with various equations with a joint density, which we assume to be a normal distribution. Because disturbances are likely to be cross-equation-correlated, Zellner’s seemingly unrelated regression method is used for estimation of the parameters of the model defined in (7) and (8) [63]. As usual, because the shares add up to one, causing the variance–covariance matrix of the error terms to be singular, one share equation in the direct cost function model is eliminated. After the parameters of the model have been estimated, FTCs are calculated by applying Equation (11).

**Table 6:** Estimates translog cost function model (*N* = 682)

Variable	Estimate	St. error	*T* value
Constant	0.270	0.016	17.14
Admissions	0.656	0.022	29.61
Outpatients	0.383	0.024	16.14
Other revenues	0.072	0.009	7.96
Admissions * admissions	−0.160	0.069	−2.32
Admissions * outpatients	0.162	0.067	2.42
Admissions * other revenues	0.061	0.026	2.35
Outpatients * outpatients	−0.239	0.092	−2.59
Outpatients* other revenues	−0.012	0.027	−0.44
Other revenues* other revenues	−0.021	0.009	−2.30
Price management and clerical staff	0.101	0.002	42.13
Price nursing personnel	0.335	0.004	87.37
Price paramedical personnel	0.049	0.002	26.87
Price auxiliary personnel	0.109	0.002	44.37
Price material patients	0.231	0.004	51.54
Price material general	0.078	0.003	25.18
Price capital	0.097	0.002	61.26
Price man. and staff. * price man. and staff.	0.055	0.006	9.36
Price man. and staff.* price nursing personnel	0.004	0.007	0.61
Price man. and staff. * price medical personnel	−0.004	0.002	−1.92
Price man. and staff. * price auxiliary personnel	−0.010	0.002	−5.44
Price man. and staff. * price material patients	−0.030	0.008	−3.78
Price man. and staff. * price material general	−0.007	0.006	−1.13
Price man. and staff.* price capital	−0.008	0.002	−3.69
Price nursing personnel * price nursing personnel	0.088	0.015	5.98
Price nursing personnel * price medical personnel	0.011	0.003	3.26
Price nursing personnel * price auxiliary personnel	−0.023	0.003	−7.57
Price nursing personnel * price material patients	−0.021	0.014	−1.55
Price nursing personnel * price material general	−0.034	0.011	−3.14
Price nursing personnel * price capital	−0.025	0.003	−7.66
Price medical personnel * price medical personnel	0.012	0.002	5.82
Price medical personnel * price auxiliary personnel	0.003	0.001	2.10
Price medical personnel * price material patients	−0.009	0.004	−2.42
Price medical personnel * price material general	−0.010	0.003	−3.74
Price medical personnel * price capital	−0.003	0.001	−2.26
Price auxiliary personnel * price auxiliary personnel	0.026	0.002	10.90
Price auxiliary personnel * price material patients	0.006	0.003	2.09
Price auxiliary personnel * price material general	0.002	0.002	1.02
Price auxiliary personnel * price capital	−0.004	0.001	−3.17
Price medical supplies *price material patients	0.024	0.044	0.55
Price material patients * price material general	0.034	0.041	0.83
Price material patients * capital	−0.004	0.004	−1.18
Price material general * price material general	0.028	0.040	0.69
price material supplies * price capital	−0.013	0.003	−4.53
price capital * price capital	0.057	0.002	34.27
Admissions * price man. and staff.	0.000 03	0.003	0.01
Admissions* price nursing personnel	−0.018	0.004	−4.11
Admissions * price medical personnel	0.011	0.002	4.51
Admissions * price auxiliary personnel	−0.010	0.003	−3.12
Admissions * price material patients	0.031	0.005	6.82
Admissions * price material general	−0.003	0.003	−1.15
Admissions * capital	−0.009	0.002	−5.07
Outpatients * price man. and staff.	0.006	0.003	1.87
Outpatients * price nursing personnel	0.012	0.005	2.31
Outpatients * price medical personnel	0.003	0.003	1.17
Outpatients * price auxiliary personnel	0.007	0.004	1.80
Outpatients * price material patients	−0.033	0.005	−6.24
Outpatients * price material general	−0.008	0.004	−2.30
Outpatients * capital	0.012	0.002	5.87
Other rev. * price man. and staff.	−0.001	0.001	−0.47
Other rev. * price nursing personnel	−0.001	0.002	−0.59
Other rev. * price medical personnel	0.005	0.001	5.49
Other rev. * price auxiliary personnel	−0.004	0.001	−3.05
Other rev. * price material patients	0.000 5	0.002	0.27
Other rev. * price material general	0.003	0.001	2.36
Other rev. * capital	−0.003	0.001	−3.99
Time	−0.030	0.005	−6.24
Time squared	0.001	0.001	1.11
Trend * price man. and staff.	−0.000 5	0.000 3	−1.41
Trend * price nursing personnel	−0.001	0.001	−2.45
Trend * price medical personnel	−0.001	0.000 3	−2.19
Trend * price auxiliary personnel	−0.002	0.000 4	−5.60
Trend * price material patients	0.003	0.001	4.87
Trend * price material general	0.002	0.000 4	4.11
Trend * price capital	−0.001	0.000 2	−2.44
Admissions * surgery and orthopaedics	−0.158	0.026	−5.98
Admissions * psychiatric beds	0.013	0.003	3.99
Admissions * IC beds	0.018	0.011	1.67
Admissions * expected length of stay	0.241	0.063	3.80
Admissions * neurosurgery	0.028	0.004	6.57
Admissions * cardiothoracic surgery	0.098	0.010	9.64
Admissions * cardiothoracic surgery	0.140	0.012	11.920
*R* ^2^ cost function	0.98		
*R* ^2^ management and clerical staff	0.21		
*R* ^2^ nursing personnel	0.29		
*R* ^2^ paramedical personnel	0.37		
*R* ^2^ auxiliary personnel	0.30		
*R* ^2^ medical supplies	0.46		
*R* ^2^ material supplies	0.10		
*R* ^2^ capital	0.66		

## Abbreviations

FTC: Factor technical change
OECD: Organisation for Economic Co-operation and Development
TFP: Total factor productivity

## Footnotes

1 During 2003–2011, the average length of stay per admission dropped from 7 to 5 days. Including day care, the decline was even greater, with an average of 4.5 days in 2003 and 2.9 days in 2011.
